# Single Nucleotide Variants in the Protein C Pathway and Mortality in Dialysis Patients

**DOI:** 10.1371/journal.pone.0097251

**Published:** 2014-05-09

**Authors:** Gürbey Ocak, Christiane Drechsler, Carla Y. Vossen, Hans L. Vos, Frits R. Rosendaal, Pieter H. Reitsma, Michael M. Hoffmann, Winfried März, Willem H. Ouwehand, Raymond T. Krediet, Elisabeth W. Boeschoten, Friedo W. Dekker, Christoph Wanner, Marion Verduijn

**Affiliations:** 1 Department of Clinical Epidemiology, Leiden University Medical Center, Leiden, The Netherlands; 2 Department of Medicine, Division of Nephrology, University Hospital, Würzburg, Germany; 3 Comprehensive Heart Failure Center, University of Wuerzburg, Wuerzburg, Germany; 4 Institute of Clinical Epidemiology and Biometry, University of Wuerzburg, Wuerzburg, Germany; 5 Department of Thrombosis and Haemostasis, Leiden University Medical Center, Leiden, The Netherlands; 6 Einthoven Laboratory for Experimental Vascular Medicine, Leiden University Medical Center, Leiden, The Netherlands; 7 Institute of Clinical Chemistry and Laboratory Medicine, University Medical Center, Freiburg, Germany; 8 Department of Public Health, Social and Preventive Medicine, University of Heidelberg, Mannheim, Germany; 9 Department of Hematology, University of Cambridge and National Health Service Blood and Transplant, Cambridge, United Kingdom; 10 Department of Human Genetics, Wellcome Trust Sanger Institute, Cambridge, United Kingdom; 11 Department of Nephrology, Academic Medical Center, Amsterdam, The Netherlands; 12 Hans Mak Institute, Naarden, The Netherlands; University of Pennsylvania, United States of America

## Abstract

**Background:**

The protein C pathway plays an important role in the maintenance of endothelial barrier function and in the inflammatory and coagulant processes that are characteristic of patients on dialysis. We investigated whether common single nucleotide variants (SNV) in genes encoding protein C pathway components were associated with all-cause 5 years mortality risk in dialysis patients.

**Methods:**

Single nucleotides variants in the factor V gene (*F5* rs6025; factor V Leiden), the thrombomodulin gene (*THBD* rs1042580), the protein C gene (*PROC* rs1799808 and 1799809) and the endothelial protein C receptor gene (*PROCR* rs867186, rs2069951, and rs2069952) were genotyped in 1070 dialysis patients from the NEtherlands COoperative Study on the Adequacy of Dialysis (NECOSAD) cohort) and in 1243 dialysis patients from the German 4D cohort.

**Results:**

Factor V Leiden was associated with a 1.5-fold (95% CI 1.1–1.9) increased 5-year all-cause mortality risk and carriers of the AG/GG genotypes of the PROC rs1799809 had a 1.2-fold (95% CI 1.0–1.4) increased 5-year all-cause mortality risk. The other SNVs in *THBD*, *PROC*, and *PROCR* were not associated with 5-years mortality.

**Conclusion:**

Our study suggests that factor V Leiden and PROC rs1799809 contributes to an increased mortality risk in dialysis patients.

## Introduction

The protein C pathway plays an important role in endothelial barrier function and in inflammatory and anticoagulant processes [1]. Protein C activation occurs on the endothelial cell membrane by thrombin bound to thrombomodulin and this is enhanced when protein C is bound to the endothelial protein C receptor. Activated protein C together with its cofactor protein S inactivates the procoagulant factors Va and VIIIa. Activated protein C resistance is often caused by a variant of factor V (factor V Leiden), that abrogates one of the inactivation sites in factor Va [2]. Besides anticoagulant properties, activated protein C has direct cytoprotective effects on endothelial cells that include anti-inflammatory actions, anti-apoptotic activities, and stabilization of endothelial barriers. These effects are largely mediated by activation of protease activated receptors [3]–[7].

The crucial role of the protein C pathway in endothelial function, coagulation, and inflammation became evident in several studies [8]–[12]. The importance of the protein C system is most clearly demonstrated by the massive thrombotic complications occurring in infants with severe protein C deficiency due to genetic abnormalities [8] and the increased risk of venous thrombosis in adults with a genetic defect resulting in decreased protein C levels [9]. In severe sepsis patients with a high mortality risk treatment with activated protein C reduced mortality, probably through its anti-inflammatory and anticoagulant activities [10]. In addition, low plasma protein C levels have been shown to increase the risk of ischemic stroke [11]. Finally, particular combinations of variants in the thrombomodulin, protein C, and factor V genes seem to increase the risk of cardiovascular events in the general population [12].

Patients on dialysis have a high mortality risk due to endothelial damage and subsequent cardiovascular diseases [13]. Dialysis patients also have a high risk of dying from dialysis treatment failure [13], which is associated with thrombotic events (i.e. vascular access thrombosis and catheter thrombosis) and infections [13]. Genetic variation in the protein C pathway could influence the mortality risk by changing processes related to endothelial damage, by influencing inflammatory response, and by increasing or decreasing the chance of thrombotic events associated with treatment failure. We hypothesized that single nucleotide variants encoding protein C pathway components or targets might influence mortality rates in dialysis patients.

We selected seven single nucleotide variants (SNVs) that are known to influence levels or activity of proteins in the protein C pathway or have been associated with venous thrombosis, arterial thrombosis or mortality in the general population: factor V (*F5*) rs6025 (factor V Leiden) [2], thrombomodulin (*THBD*) rs1042580 [12], [14], protein C (*PROC*) rs1799809 and rs1799808 [15], and protein C receptor (*PROCR*) rs867186, rs2069952, and rs2069952.^16^ We investigated the association between these SNVs and all-cause and cause-specific (cardiovascular and non-cardiovascular) mortality in the NEtherlands COoperative Study on the Adequacy of Dialysis (NECOSAD) cohort and the German Diabetes Dialysis Study (4D-study).

## Methods and Materials

### Patients

The Netherlands Cooperative Study on the Adequacy of Dialysis (NECOSAD) is a prospective multicenter cohort study in which incident adult dialysis patients from 38 dialysis centers in the Netherlands were included. The Medical Review Ethics Committee of the Leiden University Medical Center approved the study. All patients gave written informed consent. Eligibility criteria included age older than 18 years, and no previous renal replacement therapy (transplantation or dialysis). For the current analyses, we used data from patients who were included between June 1997 and June 2007 in 23 dialysis centers that approved DNA analysis. Information was gathered from patients until date of death or date of censoring, i.e. transfer to a nonparticipating dialysis center, withdrawal from the study, transplantation, or end of the follow-up period in June 2009, whichever occurred first.

### Demographic and clinical data

Data on age, sex, primary kidney disease, and cardiovascular disease were collected at the start of dialysis treatment. Pre-existing cardiovascular disease was defined as a history of angina pectoris, myocardial infarction, heart failure, ischemic stroke, or claudication at the time of inclusion.

### Single nucleotide variants

Blood samples were collected for DNA analysis. We genotyped one SNV in the factor V gene (*F5* rs6025; factor V Leiden), one SNV in the thrombomodulin gene (*THBD* rs1042580), two SNVs in the protein C gene (*PROC* rs1799809 and rs1799808), and three SNVs in the protein C receptor gene (*PROCR* rs867186, rs2069951, and rs2069952) using TaqMan SNV Genotyping Assays (Applied Biosystems, Foster City, CA, USA) as described previously [14]–[17].

### Mortality

We classified causes of death according to the codes of the European Renal Association-European Dialysis and Transplantation Association (ERA-EDTA) which is a standardized classification of death causes in dialysis patients [18]. We grouped death causes into cardiovascular and non-cardiovascular mortality. Cardiovascular mortality was defined as death due to myocardial ischemia and infarction (code 11); cardiac arrest/sudden death (code 15); cardiac failure/fluid overload/pulmonary edema (codes 14,16,18); hyperkalemia/hypokalemia (code 12,17); pulmonary embolism (code 21); cerebrum-vascular accident (code 22); hemorrhage from ruptured vascular aneurysm (code 26); mesenteric infarction (code 29); cause of death uncertain/unknown (code 0). All other deaths were designated as non-cardiovascular mortality.

### Replication

For independent replication of the results of the NECOSAD study, we analyzed data from the German Diabetes Dialysis Study (4D-study). Methods of the 4D-study have been described in detail previously [19]. Briefly, the 4D-study was a double-blind, randomized trial on the effect of atorvastatin in hemodialysis patients with type 2 diabetes mellitus who had less than two years of previous hemodialysis treatment. The primary endpoint of was a composite of cardiac death, non-fatal myocardial infarction and stroke, whichever occurred first. Patients were randomly assigned to either 20 mg of atorvastatin or placebo once daily until the date of death, censoring, or the end of study in March 2004. Atorvastatin showed no effect on the composite primary end point [19]. The genotyping of the SNV in the factor V gene (*F5* rs6025; factor V Leiden), the SNV in the thrombomodulin gene (*THBD* rs1042580), the two SNVs in the protein C gene (*PROC* rs1799809 and rs1799808), and the three SNVs in the protein C receptor gene (*PROCR* rs867186, rs2069951, and rs2069952) were the same as described above for the NECOSAD cohort. The SNV in the factor V gene (*F5* rs6025; factor V Leiden), the SNV in the thrombomodulin gene (*THBD* rs1042580), and the SNV in the protein C receptor gene (*PROCR* rs867186) were genotyped earlier than the other SNVs, therefore the numbers vary for these SNVs as compared with the other SNVs. Mortality was also categorized into cardiovascular and non-cardiovascular deaths.

### Statistical analysis

The baseline characteristics are presented as median and 5^th^–95^th^ percentiles for continuous variables, and as percentages for categorical variables. Distributions of genotypes were compared by the chi-square test to test for Hardy-Weinberg equilibrium. We calculated pooled hazard ratios (HRs) with 95% confidence intervals (95% CIs) for all-cause, cardiovascular, and non-cardiovascular mortality by Cox's regression analysis to study the effect of the seven SNVs on 5-year mortality from start of dialysis in the NECOSAD and 4D-study together. Moreover, we investigated the effect on mortality of combinations of *THBD* rs1042580 and single nucleotide variants that increased the mortality risk of dialysis patients in our study, since a previous study found associations between the combination of single nucleotide variants in the protein C pathway and THBD rs1042580 in the risk of cardiovascular diseases [12]. Furthermore, we calculated HRs with 95% CIs separately for the NECOSAD cohort and 4D-study. In addition, we repeated the analyses in the NECOSAD cohort for only hemodialysis patients with diabetes mellitus, since the 4D-study consists of only hemodialysis patients with diabetes mellitus. HRs were calculated for homozygous or heterozygous carriers of the rare alleles (except for rs2069952 for which the risk allele was the common major allele) of the SNVs compared to non-carriers. We reported unadjusted HRs, since adjustment in genetic association studies could potentially introduce interference in the causal pathway and thereby bias through overadjustment [20]. We used SPSS statistical software (version 17.0; SPSS, Chicago) for all statistical analyses.

## Results

A total of 1070 patients from the NECOSAD cohort and 1243 patients from the 4D cohort were genotyped for the seven SNVs. Baseline characteristics of the 1070 patients from the NECOSAD cohort and 1243 patients from the 4D cohort are shown in Table 1. In contrast to the NECOSAD cohort, the 4D cohort consisted only of hemodialysis patients with diabetes mellitus. In the NECOSAD cohort, 140 hemodialysis patients (20.7%) had diabetes mellitus.

**Table 1:** Baseline characteristics.

		NECOSAD N = 1070		4D-study N = 1243	
Age (years), median (5^th^-95^th^ percentile)		62.2	(33.0–80.2)	66.0	(51.0–78.0)
Males, %		62.9		54.1	
Body mass index (kg/m^2^), median (5^th^–95^th^ percentile)		24.3	(19.2–33.2)	26.7	(20.7–36.3)
Dialysis duration (months), median (5^th^–95^th^ percentile)		0		6.0	(1.1–22.5)
Dialysis modality,%					
	Hemodialysis	63.4		100	
	Peritoneal dialysis	36.6		0	
History of diabetes mellitus, %		20.7		100	
Cardiovascular disease,%		35.2		29.5	

Table 2 shows the genotype and allele frequencies for the seven SNVs. All SNVs in the NECOSAD cohort were in Hardy-Weinberg equilibrium, except *PROC* rs1799809 (p-value 0.002). In the 4D cohort, *F5* rs6025 (factor V Leiden) (p-value<0.001), *PROC* rs1799808 (p-value <0.036), and *PROCR* rs2069952 (p-value 0.001) were not in Hardy-Weinberg equilibrium (Table 2).

**Table 2:** Distribution of single nucleotide variants.

			NECOSAD			4D-STUDY		
			N = 1070			N = 1243		
Gene	SNV	Genotype	N	%	HW	N	%	HW
	Location				equilibrium			equilibrium
Factor V (Leiden)	rs6025	GG	984	(94.7)	*p* = 0.157	837	(94.2)	*p*<0.001
	exon	AG	53	(5.1)		48	(5.4)	
		AA	2	(0.2)		4	(0.4)	
		Allele frequency A		2.7%			3.1%	
Thrombomodulin	rs1042580	AA	404	(38.8)	*p* = 0.443	321	(36.1)	*p* = 0.397
	3′UTR	AG	479	(46.1)		437	(49.1)	
		GG	157	(15.1)		132	(14.8)	
		Allele frequency G		38.1%			39.4%	
Protein C	rs1799808	CC	431	(41.1)	*p* = 0.681	491	(39.7)	*p* = 0.036
	promoter	CT	478	(45.6)		604	(48.8)	
		TT	140	(13.3)		143	(11.6)	
		Allele frequency T		36.1%			35.9%	
Protein C	rs1799809	AA	368	(35.2)	*p* = 0.002	402	(32.5)	*p* = 0.363
	promoter	AG	461	(44.3)		620	(50.1)	
		GG	215	(20.6)		215	(17.4)	
		Allele frequency G		42.7%			42.4%	
Protein C receptor	rs867186	AA	834	(79.8)	*p* = 0.084	667	(75.1)	*p* = 0.136
	exon	AG	193	(18.5)		211	(23.8)	
		GG	18	(1.7)		10	(1.1)	
		Allele frequency G		11.0%			13.0%	
Protein C receptor	rs2069951	GG	919	(87.1)	*p* = 0.549	1129	(91.1)	*p* = 0.372
	intron	GA	130	(12.3)		106	(8.6)	
		AA	6	(0.6)		4	(0.3)	
		Allele frequency A		6.7%			4.6%	
Protein C receptor	rs2069952	CC	164	(15.8)	*p* = 0.798	263	(21.2)	*p* = 0.001
	intron	CT	493	(47.4)		552	(44.6)	
		TT	383	(36.8)		424	(34.2)	
		Allele frequency C		39.5%			43.5%	

Of the 1070 patients from the NECOSAD cohort, 401 died within 5 years of follow-up; 185 patients due to cardiovascular causes and 216 due to non-cardiovascular causes. In the 4D cohort, 594 patients died within 5 years of follow-up (297 patients due to cardiovascular causes and 297 due to non-cardiovascular causes).

### Factor V (Leiden) rs6025

Factor V Leiden was associated with a 1.5-fold (95% CI 1.1–1.9) increased year all-cause mortality risk in the pooled results. The hazard ratios were 1.4 (95% CI 0.9–2.1) in the total NECOSAD cohort and 1.6 (95% CI 1.1–2.2) in the 4D-study (Table 3). Restricting the analyses to diabetic patients with hemodialysis in the NECOSAD study (similar to the 4D study which only includes diabetic hemodialysis patients), factor V Leiden was associated with a 2.1-fold (95% CI 1.0–4.5) increased 5-year all-cause mortality risk (Table 3).

**Table 3:** Effect of single nucleotide variants on 5-year mortality.

			POOLED		NECOSAD		NECOSAD		4D-STUDY	
			RESULTS				HD and DM*			
			N = 2313		N = 1070		N = 140		N = 1243	
Gene/SNV	Genotype	Mortality	HR	(95% CI)	HR	(95% CI)	HR	(95% CI)	HR	(95% CI)
Factor V	GG		1	(reference)	1	(reference)	1	(reference)	1	(reference)
(Leiden)/rs6025	AG/AA	All-cause	1.5	(1.1–1.9)	1.4	(0.9–2.1)	2.1	(1.0–4.5)	1.6	(1.1–2.2)
		CV	1.5	(1.0–2.2)	1.3	(0.7–2.4)	0.5	(0.1–3.9)	1.6	(1.0–2.6)
		Non-CV	1.5	(1.0–2.2)	1.4	(0.8–2.5)	4.0	(1.7–9.5)	1.6	(0.9–2.6)
Thrombomodulin/	AA		1	(reference)	1	(reference)	1	(reference)	1	(reference)
rs1042580	AG/GG	All-cause	1.0	(0.9–1.1)	1.0	(0.8–1.2)	1.2	(0.8–1.9)	1.0	(0.8–1.2)
		CV	1.0	(0.8–1.2)	0.9	(0.7–1.2)	1.0	(0.5–1.9)	1.0	(0.8–1.3)
		Non-CV	1.0	(0.8–1.2)	1.0	(0.8–1.4)	1.4	(0.7–2.8)	1.0	(0.8–1.3)
Protein C/	CC		1	(reference)	1	(reference)	1	(reference)	1	(reference)
rs1799808	CT/TT	All-cause	1.0	(0.9–1.2)	1.1	(0.9–1.3)	1.1	(0.7–1.7)	1.0	(0.8–1.2)
		CV	1.0	(0.8–1.2)	0.9	(0.7–1.3)	0.8	(0.4–1.5)	1.0	(0.8–1.3)
		Non-CV	1.0	(0.9–1.2)	1.2	(0.9–1.5)	1.5	(0.8–3.0)	1.0	(0.8–1.2)
Protein C/	AA		1	(reference)	1	(reference)	1	(reference)	1	(reference)
rs1799809	AG/GG	All-cause	1.2	(1.0–1.4)	1.2	(0.9–1.5)	0.9	(0.5–1.4)	1.2	(1.0–1.4)
		CV	1.2	(1.0–1.5)	1.2	(0.9–1.6)	1.0	(0.5–2.0)	1.3	(1.0–1.6)
		Non-CV	1.2	(1.0–1.4)	1.2	(0.9–1.6)	0.8	(0.4–1.5)	1.1	(0.9–1.5)
Protein C	AA		1	(reference)	1	(reference)	1	(reference)	1	(reference)
receptor/	AG/GG	All-cause	1.0	(0.8–1.1)	0.9	(0.7–1.2)	1.3	(0.8–2.2)	1.0	(0.8–1.2)
rs867186		CV	0.8	(0.6–1.0)	0.7	(0.5–1.0)	1.0	(0.4–2.1)	0.8	(0.6–1.1)
		Non-CV	1.2	(1.0–1.5)	1.2	(0.9–1.6)	1.7	(0.9–3.4)	1.2	(0.9–1.5)
Protein C	GG		1	(reference)	1	(reference)	1	(reference)	1	(reference)
receptor/	AG/AA	All-cause	1.1	(0.9–1.4)	1.1	(0.8–1.5)	1.0	(0.5–1.9)	1.2	(0.9–1.6)
rs2069951		CV	1.2	(0.9–1.5)	1.0	(0.7–1.6)	1.3	(0.5–3.1)	1.4	(0.9–1.9)
		Non-CV	1.1	(0.8–1.4)	1.2	(0.8–1.7)	0.6	(0.2–2.0)	1.0	(0.7–1.5)
Protein C	CC		1	(reference)	1	(reference)	1	(reference)	1	(reference)
receptor/	CT/TT	All-cause	1.0	(0.8–1.1)	0.9	(0.7–1.2)	2.3	(1.2–4.5)	1.1	(0.9–1.3)
rs2069952		CV	1.0	(0.8–1.2)	0.9	(0.6–1.3)	1.5	(0.7–3.4)	1.1	(0.8–1.4)
		Non-CV	1.0	(0.8–1.2)	1.0	(0.7–1.4)	4.1	(1.3–13.4)	1.0	(0.8–1.4)

*hemodialysis patients with diabetes mellitus

As compared with *THBD* rs1042580 AA genotype in the absence of factor V Leiden, the combination of factor V Leiden and *THBD* rs1042580 AA genotype resulted in a 1.5-fold (95% CI 0.9–2.4) increased mortality risk, the combination of *THBD* rs1042580 AA genotype in the absence of factor V Leiden resulted in a 1.0-fold (95% CI 0.9–1.1) increased mortality risk, and the combination of factor V Leiden and *THBD* rs1042580 AG/GG genotypes resulted in a 1.4-fold (95% CI 1.0–2.0) mortality risk.

### Thrombomodulin, protein C and protein C receptor variants

As compared to the AA genotype in *PROC* rs1799809, carriers of the AG/GG genotypes had a 1.2-fold (95% CI 1.0–1.4) increased 5-year all-cause mortality risk (Table 3). As compared with *PROC* rs1799809 AA genotype and *THBD* rs1042580 AA genotype, the combination of *PROC* rs1799809 AG/GG genotypes and *THBD* rs1042580 AA genotype resulted in a 1.1-fold (95% CI 0.9–1.4) increased mortally risk, the combination of *PROC* rs1799809 AA genotype and *THBD* rs1042580 AG/GG genotypes resulted in a 0.9-fold (95% CI 0.7–1.2) increased mortally risk, and the combination of *PROC* rs1799809 AG/GG genotype and *THBD* rs1042580 AG/GG genotype resulted in a 1.2-fold (95% CI 0.9–1.5) increased mortally risk.

*PROC* rs1799809, *THBD* rs1042580, *PROC* rs1799808, *PROCR* rs867186, *PROCR* rs2069951, and *PROCR* rs2069952 were not associated with all-cause mortality in the NECOSAD and the 4D cohorts (Table 3).

## Discussion

This candidate-gene study assessed the 5-years mortality risk while on dialysis treatment for seven genetic variants that influence levels or activity of proteins in the protein C pathway: one SNV in the factor V gene (factor V Leiden), two SNVs in the protein C gene (*PROC*), one SNV in the thrombomodulin gene (*THBD*) and three SNVs (tagging three haplotypes) in the protein C receptor gene (*PROCR*). We found that factor V Leiden was associated with a 1.5-fold (95% CI 1.1–1.9) increased 5-year all-cause mortality risk and that *PROC* rs1799809 was associated with a 1.2-fold (95% CI 1.0–1.4) increased 5-year all-cause mortality risk. Furthermore, we showed that *THBD* rs1042580, *PROC* rs1799808, *PROCR* rs867186, *PROCR* rs2069951, and *PROCR* rs2069952 were not associated with an increased mortality risk.

Studies in the general population have shown an association between factor V Leiden and an increased risk of different adverse outcomes, including venous thrombosis [17], ischemic stroke [21], and myocardial infarction [22]. We showed in the current study that factor V Leiden was associated with increased all-cause mortality in dialysis patients. Other studies did not find an increased all-cause mortality risk for factor V Leiden in the general population and in thrombophilic families [23], [24]. However, it could be the interaction between dialysis and factor V Leiden that could lead to an increased mortality, which is not present in the general population. Previous studies on factor V in the dialysis population have been focused on arteriovenous access failure. A recent study showed that a factor V gene SNV (rs6019) was associated with arteriovenous graft failure in dialysis patients suggesting an association between factor V SNVs and adverse outcomes in dialysis patients [25], which is in line with our study.

Factor V Leiden represents a single nucleotide variant in the Factor V gene, encoding a change in the protein from an arginine at position 506 to a glutamine. Since this amino acid is normally the cleavage site for activated protein C, the mutation prevents efficient inactivation of factor V. When factor V remains active, it facilitates overproduction of thrombin leading to a procoagulant state. Several mechanisms might provide plausible explanations for the higher mortality risk in dialysis patients associated with factor V Leiden. First, factor V Leiden in combination with pre-existing and highly prevalent endothelial damage could lead to excess mortality from cardiovascular events. Second, factor V Leiden has been associated with venous thrombosis [14], [16], [17], [26]. The excess mortality could have been caused by fatal pulmonary embolisms due to the procoagulant changes due to factor V Leiden in combination with the start of dialysis which is also associated with an increased risk of venous thrombosis [27], [28]. Arguing against this explanation is that in our study confirmed pulmonary embolism was the cause of death in only three patients, but pulmonary embolisms as cause of death might have gone undetected or misclassified as for example sudden cardiac death. Third, one of the main complications in dialysis therapy is clot formation and thrombosis in vascular accesses [29]. Factor V Leiden is associated with procoagulant changes and could therefore lead to treatment failure in dialysis patients. Previous studies have reported an increased risk of arteriovenous access failure in patients with factor V SNVs [30], [31].

*THBD* rs1042580 AG/GG genotypes have been associated with venous thrombosis in the general population [14]. In addition, the combination of *THBD* rs1042580 with different Factor V SNVs was associated with an increased risk of cardiovascular events [12]. Other *THBD* SNVs have also been associated with cardiovascular outcomes when combined with *PROC* SNVs or factor V Leiden in the general population [12]. However, we did not find an association between *THBD* rs1042580 AG/GG genotypes and mortality in dialysis patients. Furthermore, we did not find an important interaction between *THBD* rs1042580 and factor V Leiden and between *THBD* rs1042580 and *PROC* rs1799809.

In contrast to previous studies in sepsis patients, we found no association between the *PROC* rs1799808 and mortality. However, we found that *PROC* rs1799809 was associated with a small increased (hazard ratio 1.2, 95% CI 1.0–1.4) mortality risk. Haplotypes tagged by these SNVs have been associated with decreased survival and increased organ dysfunction in severe sepsis patients [32], [33]. An earlier study found that *PROC* rs1799808 was less important in the determination of protein C levels, indicating that the effect on protein C levels was mainly mediated by the *PROC* rs1799809 [15]. Studies in the general population on *PROCR* rs867186, *PROCR* rs2069951, and *PROCR* rs2069952 have been inconsistent in the risk of venous thrombosis [15], [16], [26] and arterial thrombosis [34], [35]. We did not find an association between these SNVs and mortality.

The genotype distribution of *PROC* rs1799809 deviated from Hardy-Weinberg equilibrium in the NECOSAD study and the genotype distribution of *F5* rs6025 (factor V Leiden), *PROC* rs1799808, and *PROCR* rs2069952 were not in Hardy-Weinberg equilibrium in the 4D cohort. It is likely that in diseased populations, such as dialysis patients, selection could have resulted in deviations from Hardy-Weinberg equilibrium.

A potential limitation of our study is that we replicated our results in a dialysis population consisting of hemodialysis patients with diabetes mellitus. For most of the SNVs this was not a problem, since there were no large differences when we restricted the NECOSAD cohort to hemodialysis patients with diabetes mellitus. However, in the 4D-study, we could not investigate the association between mortality and the protein C SNVs in peritoneal dialysis patients and in patients without diabetes mellitus. Furthermore, although we included more than 2000 dialysis patients, our study could be underpowered to detect small differences. In addition, we did not correct for multiple testing, since in this candidate gene study the single nucleotide were chosen based on a priori hypotheses.

In conclusion, our study suggests that factor V Leiden and *PROC* rs1799809 contributes to an increased mortality risk in dialysis patients. This study is the first to investigate the association between protein C pathway SNVs and mortality in large cohorts of dialysis patients.
